# Membranes and Membrane Reactors for Gas Purification and Production: Towards More Sustainable Processes

**DOI:** 10.3390/membranes16040150

**Published:** 2026-04-17

**Authors:** Pasquale Francesco Zito

**Affiliations:** National Research Council—Institute on Membrane Technology “Enrico Drioli” (CNR—ITM), Via P. Bucci, 17/C, 87036 Rende, CS, Italy; p.zito@itm.cnr.it

## 1. Introduction

The use of membranes for gas purification and production is becoming increasingly common as a valid alternative to the conventional technologies (i.e., absorption, cryogenic distillation, traditional reactors, etc.). Low energy consumption, small equipment size, and high yields are the main advantages that make membrane technology an attractive and sustainable option for the treatment of multicomponent mixtures in the field of H_2_ production, biogas upgrading, CO_2_ capture and so on.

In this Special Issue, several papers concerning the latest advancements in the field of membrane technology for gas separation and production have been collected.

## 2. Summary of the Special Issue Content

A total of nine papers have been included in this Special Issue, related to the use of membranes as reactors and separators. In the field of H_2_ purification, Mohtashamifar et al. investigated the possibility of reducing the Pd content in membranes by replacing it with other elements. Their analysis showed that VPd membranes are good candidates for H_2_ separation due to the lower environmental impact than PdAg membranes. Prenesti et al. studied the solubility of H_2_ in different metal-alloy membranes to identify the most appropriate materials for H_2_ purification and the embrittlement threshold due to its concentration inside the metal lattice. Regarding membrane reactors, Barmaki et al. investigated H_2_ production from methanol steam reforming via a simulation analysis, comparing the performance of fluidized and packed-bed membrane reactors with that of the conventional equipment. The authors found that fluidized-bed membrane reactors are able to ensure better performance than the other configurations. Småbråten et al. analysed H_2_ and NH_3_ co-adsorption on several metal surfaces (i.e., Pd, Ru, Ag, Au, Cu), finding that Pd and Pd alloys show the best surface H_2_ adsorption properties, whereas the presence of a large amount of the other metals in the alloys can be damaging. Concerning the CO_2_/CH_4_ separation, Li et al. synthesised ester-crosslinked polyimide membranes with interesting separation performance and a high plasticization resistance, up to pressure higher than 30 bar. Kuznetsova et al. compared the performance of zeolite and polymeric membranes for natural gas upgrading, finding that zeolite membranes exhibit the simplest design and the lowest cost, as they can achieve the purification target in a single stage. CO_2_ capture strategies were also investigated by Jin et al., who developed a non-isothermal mathematical model to simulate and predict the CO_2_ absorption by three absorbents in a hollow fibre membrane contactor. Finally, in the field of H_2_/CH_4_ separation, Yi et al. analysed the permeation through MFI membranes activated by ozonation, achieving a selectivity of 23.8 at 100 °C. These membranes were also found to be suitable for IPA dehydration by pervaporation. A techno-economic analysis on different membrane configurations for H_2_ and CH_4_ purification was conducted by Zito, pairing Pd-Ag with zeolite membranes. This assessment demonstrated the potential of multistage processes to generate profit, even if the single-stage Pd-Ag membrane plant resulted in the most cost-effective solution.

## 3. Conclusions

This Special Issue dealt with several aspects of gas purification and production using membrane processes. The contributions reported here highlight the role of membrane technology in developing processes oriented toward process intensification, lower greenhouse gas emissions and higher use of alternative sources to fossil fuels.

